# Effectiveness of mindful breathing exercises on symptom reduction and quality of life in individuals with major depressive disorder: A systematic review

**DOI:** 10.4102/sajp.v82i1.2352

**Published:** 2026-05-22

**Authors:** Saad Bin Muaythir, Nombeko Mshunqane, Enos Ramano, Bashir Bello, Nontembiso Magida

**Affiliations:** 1 Department of Physiotherapy, Faculty of Health Sciences, University of Pretoria, Pretoria, South Africa; 2 Department of Physiotherapy, Faculty of Health, University of KwaZulu-Natal, Durban, South Africa; 3 Department of Occupational Therapy, University of Pretoria, Pretoria, South Africa; 4 Department of Physiotherapy, Faculty of Health, Bayero University, Kano, Nigeria

**Keywords:** mindful breathing, major depressive disorder, quality of life, symptom reduction, systematic review, breathing exercises

## Abstract

**Background:**

Major depressive disorder (MDD) is a global cause of disability, with many patients experiencing suboptimal response to conventional treatments. Mindful breathing exercises, a simple and scalable technique, have emerged as a promising non-pharmacological intervention for alleviating depressive symptoms and improving quality of life.

**Objectives:**

To evaluate the effectiveness of mindful breathing exercises on symptom reduction and quality of life in individuals diagnosed with MDD.

**Method:**

A systematic search of Cochrane Library, MEDLINE (PubMed), PEDro, Index to Nursing and Allied Health Literature (CINAHL), Scopus, Web of Science and BioMed Central databases was conducted from inception to September 2025. Our review was conducted in accordance with the Preferred Reporting Items for Systematic Reviews and Meta-Analyses guidelines. Eligible criteria were intervention studies examining the effects of mindful breathing on depressive symptoms and quality of life in individuals diagnosed with MDD. Two reviewers independently screened the studies, extracted the data and assessed the methodological quality using the Cochrane risk of bias tool. A narrative synthesis was conducted because of heterogeneity in outcome measures and intervention protocols.

**Results:**

Two studies involving 179 participants met the inclusion criteria. One study combined cognitive behavioural therapy with breathing exercises, demonstrating improvements in sleep quality, psychiatric symptoms and heart rate variability. Another study demonstrated a significant reduction in anxiety.

**Conclusion:**

Mindful breathing exercises are beneficial in alleviating symptoms of depression and anxiety, as well as enhancing physiological indicators in people with MDD.

**Clinical Implications:**

Our review highlights breathing techniques as an affordable, non-pharmaceutical option to conventional treatment; however, more research with rigorous outcomes is needed.

## Introduction

Major depressive disorder (MDD) is a debilitating mental health condition characterised by persistent low mood, anhedonia, cognitive impairment, fatigue and diminished capacity for daily functioning (Regier, Kuhl & Kupfer 2013). Globally, MDD affects more than 280 million people, representing a significant contributor to the overall burden of disease and the leading cause of disability worldwide (World Health Organization [WHO] 2023). Despite the availability of pharmacological and psychotherapeutic treatments, many individuals with MDD experience suboptimal outcomes, relapse or treatment resistance, thereby prompting the exploration of complementary approaches to enhance recovery and well-being (Cuijpers et al. 2014).

In recent years, there has been increasing interest in mindfulness-based interventions, particularly mindful breathing exercises, as adjunctive strategies for managing depressive symptoms. Mindful breathing is a core component of mindfulness practice, involving the intentional direction of attention to the natural rhythm of the breath while cultivating non-judgemental awareness of thoughts, feelings and bodily sensations (Kabat-Zinn & Hanh 2009). This practice is thought to promote emotional regulation, reduce rumination and enhance cognitive flexibility, key mechanisms implicated in the pathophysiology and maintenance of depression (Gu et al. 2015; Segal, Williams & Teasdale 2012). Empirical studies have demonstrated that mindful breathing may yield beneficial effects on mental health by modulating activity in neural circuits related to stress response, attention and self-referential processing (Weder 2022). Furthermore, breathing-focused interventions are easily teachable, cost-effective and can be delivered in various formats, including individual, group and digital platforms, thereby enhancing accessibility and scalability in diverse clinical and community settings (Goyal et al. 2014; Spijkerman, Pots & Bohlmeijer 2016).

While research has consistently shown the therapeutic advantages of mindful breathing and similar mindfulness-based practices in alleviating depressive symptoms (Teasdale et al. 2000), recent studies continue to emphasise the extensive benefits of mindfulness-based interventions for mood management and emotional health (Goldberg et al. 2018; Perestelo-Perez et al. 2017).

Nevertheless, in spite of an increasing interest in mindfulness exercises, there is still a significant lack of recent research (2020–2024) that investigates mindful breathing as a unique intervention specifically for individuals with MDD. The latest systematic reviews have concentrated mainly on general mindfulness programmes or mindfulness-based cognitive therapy rather than on focused breathing techniques (Gkintoni, Vassilopoulos & Nikolaou 2025; Knep & Shires 2025).

Although several reviews have explored the broader effects of mindfulness or mindful breathing intervention on depression, there remains a gap in the literature regarding the specific role of mindful breathing exercises as an independent or primary modality for symptom reduction and quality of life improvement in individuals diagnosed with MDD. Existing evidence is fragmented, with variations in intervention protocols, outcome measures and population characteristics, making it difficult to draw clear conclusions about efficacy (Khoury et al. 2013; Strauss et al. 2014). From a physiotherapy standpoint, practising mindful breathing is an affordable, non-drug approach that can easily be incorporated into standard procedures to promote both physical and mental health, tackling stress, regulating the autonomic nervous system and improving functional results. Assessing its effectiveness provides physiotherapists with evidence-based methods to improve comprehensive patient care in mental health environment settings.

Given the growing adoption of mindfulness-based strategies in mental health care, it is imperative to systematically assess and synthesise the current evidence on the effectiveness of mindful breathing exercises for individuals with MDD. Our review aims to evaluate the impact of mindful breathing exercises on depressive symptoms and quality of life in individuals with major depressive disorder. Accordingly, our review seeks to answer the following research question: What is the impact of mindful breathing exercises on depressive symptoms and quality of life in adults diagnosed with major depressive disorder?

## Research methods and design

### Aim

This systematic review was conducted in accordance with the Preferred Reporting Items for Systematic Reviews and Meta-Analyses (PRISMA) guidelines (Moher et al. 2009). Our review protocol was registered with PROSPERO, ensuring the transparency and reproducibility of our review process. PROSPERO registration ID: CRD420251032845. The methodological framework followed a structured approach to identify, screen and synthesise evidence on the effectiveness of mindful breathing exercises on depressive symptoms severity and quality of life for individuals diagnosed with MDD.

### Search strategy

A comprehensive literature search was performed across Cochrane Library, MEDLINE (via PubMed), PEDro, Index to Nursing and Allied Health Literature (CINAHL), Scopus, Web of Science and BioMed Central databases. The search included studies published from inception to September 2025. Search terms combined Medical Subject Headings (MeSH) and free-text terms related to ‘mindful breathing’, ‘breathing exercises’, ‘major depressive disorder’, ‘depression’, ‘quality of life’ and ‘symptom reduction’. Boolean operators (AND, OR) were used to refine the search. Additional articles were identified through reference lists of eligible studies and relevant reviews.

### Eligibility criteria

Studies were included if they investigated the effects of mindful breathing exercises, either as an independent intervention or as a major component of a mindfulness-based protocol, on individuals diagnosed with MDD and assessed depressive symptoms, quality of life and other health indices.

Our review considered intervention studies. Only peer-reviewed articles in English were included. Studies focusing exclusively on other mindfulness elements, such as body scan or yoga, without a breathing component, were excluded.

### Study selection

**FIGURE 1:**
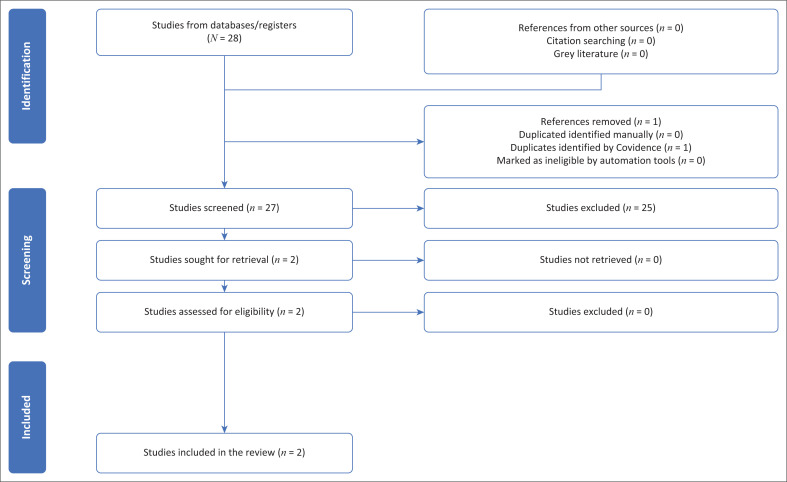
A flowchart of the screening and selection process.

All identified citations were imported into Covidence software (Veritas Health Innovation Ltd, Melbourne, Australia) for reference management and de-duplication. Titles and abstracts were independently screened by three reviewers (Saad Bin Muaythir, Enos Ramano and Bashir Bello) to identify potentially eligible studies. Full-text articles were then assessed for inclusion based on the predefined criteria. Any discrepancies were resolved through discussion or consultation with a third reviewer (Nontembiso Magida). Figure 1 demonstrates the flowchart of the screening and selection process.

### Data extraction

A standardised data extraction tool was developed and piloted. The extracted data included: authors, publication year, study design, sample size, participant characteristics (age, sex, clinical diagnosis), type and duration of mindful breathing intervention, outcome measures, key findings on symptom reduction and quality of life (Table 1). Extraction was conducted independently by three reviewers (Saad Bin Muaythir, Nombeko Mshunqane and Bashir Bello) to ensure accuracy and completeness.

**TABLE 1:** Characteristics and results of the extracted data.

Authors	Study design	Sample size (*n*)	Participant characteristics	Type and duration of mindful breathing	Outcome measures	Key findings	Recommendations
Chien et al. (2015)	Randomised controlled trial with repeated measures	89	Mean age 28.5 ± 11 years; 94.4% male; DSM-IV diagnosis of major depression	Breathing relaxation exercise; 10 min/session, 3x/week for 4 weeks (12 sessions)	Sleep quality, Heart rate variability, Brief Psychiatric Rating Scale	Significant improvements in sleep quality and heart rate variability; reduced psychiatric symptoms; effects sustained at follow-up	Use cognitive behavioural intervention and breathing exercises during hospitalisation for depression; replicate in chronic care settings
Moghadam et al. (2022)	Randomised controlled clinical trial	90	Age 18–60 years; balanced gender; clinical diagnosis of depression; undergoing electroconvulsive therapy	Slow breathing (6 breaths/min); 10 min before electroconvulsive therapy session	Anxiety (Beck Anxiety Inventory)	Significant anxiety reduction post-intervention in the breathing group (*p* < 0.001)	Breathing exercises are simple, cost-effective and useful pre-electroconvulsive therapy interventions

Note: Please see the full reference list of this article, Muaythir, S.B., Mshunqane, N., Ramano, E., Bello, B. & Magida, N., 2026, ‘Effectiveness of mindful breathing exercises on symptom reduction and quality of life in individuals with major depressive disorder: A systematic review’, South African Journal of Physiotherapy 82(1), a2352. https://doi.org/10.4102/sajp.v82i1.2352, for more information.

DSM-IV, diagnostic and statistical manual of mental disorders, 4th Edition.

### Quality assessment

The methodological quality of included randomised controlled trials was assessed using the Cochrane Risk of Bias tool (RoB) (Higgins et al. 2011) (Table 2). The risk of bias assessments was performed independently by two reviewers (Enos Ramano and Nombeko Mshunqane), and no disagreements were encountered in the process.

**TABLE 2:** Risk of bias assessment using the Cochrane Collaboration’s tool.

Study	Random sequence generation bias	Allocation concealment bias	Blinding of participants and personnel bias	Blinding of outcome assessment bias	Incomplete outcome data bias	Selective reporting (reporting bias)	Other sources of bias	Overall risk of bias
Chien et al. (2015)	Low risk	Unclear risk	High risk	High risk	Low risk	Low risk	Low risk	Unclear risk (Moderate quality: [12/21])
Moghadam et al. (2022)	Low risk	Low risk	High risk	High risk	Low risk	Low risk	Low risk	Unclear risk (Moderate quality: [11/21])

Note: Please see the full reference list of this article, Muaythir, S.B., Mshunqane, N., Ramano, E., Bello, B. & Magida, N., 2026, ‘Effectiveness of mindful breathing exercises on symptom reduction and quality of life in individuals with major depressive disorder: A systematic review’, South African Journal of Physiotherapy 82(1), a2352. https://doi.org/10.4102/sajp.v82i1.2352, for more information.

### Data synthesis

Given the heterogeneity of interventions and outcome measures, a narrative synthesis approach was adopted. Studies were grouped by intervention type and outcome category (symptom severity and quality of life). Results were described in terms of the direction and magnitude of effect. Because of the limited availability of comparable quantitative data, meta-analysis was not conducted.

### Ethical considerations

Ethical clearance to conduct this study was obtained from the University of Pretoria Faculty of Health Sciences Research Ethics Committee (No. 680/2024).

## Results

Two randomised controlled trials met the eligibility criteria and were included in our review (Table 1). Both studies evaluated the effectiveness of mindful breathing interventions in reducing symptoms in individuals diagnosed with MDD. While differing in design emphasis and outcomes, both demonstrated measurable clinical benefits. However, neither study provided a comprehensive assessment of quality-of-life outcomes, which typically include psychological well-being, social functioning, daily role functioning and overall life satisfaction. One study employed a randomised controlled trial with repeated measures to assess the impact of a combined cognitive behavioural intervention and breathing relaxation exercise on sleep quality and heart rate variability among 89 hospitalised psychiatric patients in Taiwan (Chien et al. 2015). The sample comprised predominantly male participants (94.4%) with a mean age of 28.5 years, all of whom had a clinical diagnosis of MDD based on Diagnostic and Statistical Manual of Mental Disorders, 4th Edition (diagnostic and statistical manual of mental disorders, 4th Edition.) criteria. The intervention consisted of 12 sessions delivered over 4 weeks (three 60-min sessions per week), with each session including 10 min of breathing relaxation. Outcome measures included the Pittsburgh Sleep Quality Index, Brief Psychiatric Rating Scale and physiological measures of heart rate variability. The results revealed statistically significant improvements in both sleep quality and autonomic regulation, as indicated by increased high-frequency and decreased low-frequency heart rate variability, sustained at follow-up. The authors recommended integrating cognitive breathing relaxation exercise into inpatient care for MDD, particularly in acute psychiatric settings (Chien et al. 2015).

Another study conducted a randomised controlled clinical trial among 90 patients undergoing electroconvulsive therapy in Iran. Participants were equally divided into three groups: aromatherapy with lavender essential oil, breathing exercises and routine care (Moghadam et al. 2022). Only data from the breathing exercise group (*n* = 30) were extracted for this review. The participants, aged 18–60 years, were clinically diagnosed with depression and had no prior exposure to electroconvulsive therapy. The breathing exercise intervention involved a 10-min session of slow, timed breathing (six breaths/min). Anxiety levels were assessed using the Beck Anxiety Inventory immediately before electroconvulsive therapy. Findings demonstrated a significant reduction in anxiety scores post-intervention in the breathing exercise group (*p* < 0.001), compared to baseline. Based on these findings, the authors advocated for the inclusion of simple and cost-effective breathing techniques as part of pre-electroconvulsive therapy preparation to alleviate procedure-related anxiety (Moghadam et al. 2022). Overall, the findings from both studies provide converging evidence that mindful breathing exercises, whether combined with psychological therapy or used as a standalone intervention, are effective in improving symptoms associated with MDD. Improvements were noted in physiological, emotional and functional outcomes, including reduced anxiety and improved sleep and autonomic regulation. These results support the integration of mindful breathing into therapeutic strategies for depressive disorders, which directly impact patients’ experiential quality of life during intensive psychiatric care.

### Risk of bias appraisal

Using the Cochrane Collaboration’s Risk of Bias tool, both studies were found to present an overall moderate risk of bias, primarily because of issues related to the blinding process (Chien et al. 2015; Moghadam et al. 2022) (Table 2). One study employed clustered randomisation to assign participants into intervention and control groups (Chien et al. 2015), while another study utilised a random number table to allocate participants into three arms (Moghadam et al. 2022). Therefore, the risk of bias in this domain was judged as low for both studies. Neither study provided sufficient details about how allocation concealment was achieved. The lack of information on whether group assignments were concealed from participants and researchers at the time of enrolment led to an unclear risk of bias in this domain for both studies.

Because of the nature of the interventions (breathing exercises and cognitive behavioural therapy-based group sessions), blinding of participants and interventionists was not feasible in either study. Neither study attempted to mitigate this limitation through placebo or attention control conditions.

This domain was assessed as having a high risk of bias for both trials. No explicit indication was made regarding blinding of outcome assessors in either study. Given that both studies included subjective outcomes such as self-reported anxiety (Back Anxiety inventory) and sleep quality (Pittsburgh sleep quality index), the lack of assessor blinding introduces a high risk of detection bias. In addition, Chien et al. (2015) excluded participants who were discharged before completing half of the sessions, while Moghadam et al. (2022) excluded two participants who failed to complete the post-intervention assessments. However, reasons for attrition were not provided, and the proportion of missing data was relatively low. This domain was judged to have a low risk of bias. Additionally, both studies reported their primary outcomes as specified in their objectives and provided statistical results for each. There was no evidence of selective outcome reporting.

Therefore, the risk of reporting bias was assessed as low. No significant issues were identified regarding baseline imbalances, protocol deviations or funding-related bias. This domain was assessed as low risk for both studies (Chien et al. 2015; Moghadam et al. 2022).

Both studies are moderate-quality randomised trials with notable concerns around blinding, a limitation common to behavioural and non-pharmacological interventions (Chien et al. 2015; Moghadam et al. 2022). These limitations may affect the internal validity of self-reported outcomes. Nonetheless, their use of validated outcome measures, appropriate statistical analysis and complete reporting of outcomes strengthens confidence in their findings. Future research in this area would benefit from incorporating blinding of outcome assessors and using objective outcome measures where possible.

## Discussion

This systematic review synthesised findings from two randomised controlled trials examining the effects of mindful breathing exercises on symptom reduction and quality of life in individuals with MDD (Chien et al. 2015; Moghadam et al. 2022). Both studies reported positive outcomes, reinforcing the clinical relevance of mindful breathing as a non-pharmacological intervention to improve psychological and physiological functioning in people living with depression (Chien et al. 2015; Moghadam et al. 2022). One study demonstrated that a structured intervention combining cognitive behavioural therapy with breathing relaxation exercises significantly improved sleep quality and heart rate variability in hospitalised patients with MDD (Chien et al. 2015). These physiological improvements indicate favourable autonomic modulation marked by increased parasympathetic activity and reduced sympathetic arousal. Poor sleep and autonomic dysregulation are well-documented correlates of depression (Baglioni et al., 2011; Kemp et al. 2010). Interventions that target both cognitive distortions and physiological arousal may contribute to symptom relief. However, the longer-term clinical impact remains uncertain, given the limited follow-up duration.

Additionally, another study focused on a more acute clinical setting, evaluating the anxiolytic effect of slow, diaphragmatic breathing exercises in patients with MDD undergoing electroconvulsive therapy (Moghadam et al. 2022). The intervention led to a statistically significant reduction in pre-electroconvulsive therapy anxiety scores, highlighting the effectiveness of breathing techniques in managing acute psychological distress. Anxiety associated with electroconvulsive therapy is a known barrier to treatment adherence and patient satisfaction (Obbels et al. 2017). Interventions like guided breathing, which are easy to implement and do not require extensive training or equipment, offer a low-cost strategy to support patients in high-stress clinical environments. However, longer-term emotional and quality-of-life outcomes were not measured.

Despite methodological differences, the core finding across both studies (Chien et al. 2015; Moghadam et al. 2022) is that mindful breathing exerts measurable psychological and physiological benefits in individuals with depression. This aligns with the broader literature, which demonstrates that breathing-based techniques reduce stress, enhance emotional regulation and improve mood symptoms across both clinical and non-clinical populations (Chen et al., 2017; Zaccaro et al. 2018). Mechanistically, slow and deliberate breathing appears to influence vagal tone, promote neurovisceral integration and enhance interoceptive awareness, all of which are implicated in the pathophysiology and treatment of depression (Critchley et al., 2004; Porges 2007).

Both studies had methodological limitations that should temper the interpretation of the findings.

The most notable concern was the lack of blinding of participants’ outcome assessors, and reliance on self-reported outcomes increases susceptibility to expectancy bias (Lauderdale et al. 2008). People with higher levels of anxiety frequently exhibit negative expectancy bias, which can impact their reporting of changes in symptoms (Tadic et al. 2014). Furthermore, intervention studies indicate that patient expectations can significantly influence self-reported anxiety results, even when there are only minor physiological changes (Colloca 2019).

Moreover, neither directly measured broader quality-of-life domains such as social functioning, daily activity or life satisfaction, limiting conclusions regarding functional recovery. Short follow-up periods further restrict conclusions regarding the sustainability of effects. While some literature suggests short-term benefits of mind–body interventions, long-term maintenance remains insufficiently studied and inconsistent across trials. Research on relapse prevention in depression and anxiety reveals that benefits may diminish without continued intervention (Krijnen-de Bruin et al. 2022).

Future research should incorporate longer follow-up periods, validated multidimensional quality-of-life measures and objective physiological or neurobiological markers to clarify durability and mechanisms. Broader population sampling across outpatient and community settings is also warranted (Smith et al. 2022). Although mind–body interventions, such as mindful breathing, show short-term decrease in depressive symptoms and enhancements in quality of life, the long-term persistence of these benefits is uncertain. A recent systematic review and meta-analysis revealed that while breathing-based interventions enhanced immediate post-intervention results, very few studies incorporated follow-up assessments, making it challenging to ascertain whether the improvements last beyond a few weeks (Mütze, Mitzinger & Haller 2025). In addition, several randomised controlled trials included in the meta-analysis were found to have a high risk of bias and considerable heterogeneity, which further diminishes confidence in the long-term effects (Wu, Yan & Yang 2023). Nevertheless, new evidence from a pilot study indicates that mind–body practices may sustain reductions in depression, stress and anxiety for up to a year, suggesting potential longevity when interventions are regularly practised (Kinser, Elswick & Kornstein 2014). In a similar vein, a recent systematic review of yoga for major depressive disorder showed short-term effectiveness but noted that a lack of follow-up data hinders definitive conclusions about lasting improvements (Wu et al. 2023). Overall, the existing evidence suggests that breathing-based interventions have encouraging immediate effects, while revealing an ongoing gap in their long-term maintenance, which necessitates further longitudinal research.

The sustainability of improvements in depression symptoms and quality of life beyond a few weeks remains uncertain. Future studies should include longer follow-up periods and incorporate validated multidimensional measures of quality of life. Moreover, expanding research to diverse settings and populations, including community-dwelling individuals, adolescents and those with treatment-resistant depression, would enhance generalisability. Despite these limitations, the findings support mindful breathing as a feasible, low-cost adjunct to conventional depression treatments. Clinicians should consider incorporating structured breathing protocols into inpatient and outpatient care plans, especially where pharmacotherapy is poorly tolerated or declined. The simplicity of mindful breathing also lends itself well to digital delivery, including mobile health apps and telehealth sessions, which could extend access in underserved areas.

Although only two randomised controlled trials met the inclusion criteria, our review proposes several important gaps and directions for future inquiry. Firstly, neither study conducted a long-term follow-up to assess whether the benefits of mindful breathing, such as reduced anxiety or improved sleep quality, were sustained over time. The short duration of the outcome assessment limits conclusions about the persistence of therapeutic effects, especially in a population where relapse is common. Secondly, the scope of the outcome measures was narrow. While both studies assessed symptom-specific improvements (sleep and anxiety), broader domains of quality of life, functional recovery and patient satisfaction were not addressed. There is also a lack of neurobiological or biomarker data that could shed light on the mechanisms through which mindful breathing affects affective symptoms. Another gap is the limited diversity of participant settings.

One study was hospital-based (Chien et al. 2015), and the other focused on patients receiving electroconvulsive therapy. Thus, there is a need for future research in outpatient, primary care and community mental health settings, particularly among populations with major depressive disorders or those with limited access to formal psychotherapy. Furthermore, neither study employed digital tools nor mobile delivery methods, which are an increasingly relevant approach in scalable mental health interventions.

Despite the small number of studies included, our review remains valuable. Systematic reviews are not solely dependent on quantity but also on the uniqueness and clinical significance of the data they synthesise. Both studies offered high-quality insights on a low-cost, underutilised strategy, mindful breathing, that can complement pharmacologic and psychotherapeutic treatments for MDD. The consistency of positive findings across different clinical contexts suggests a potential benefit worth exploring further. Moreover, the limited literature highlights the need for additional well-designed trials. By synthesising and appraising current evidence, our review provides a structured foundation and methodological direction for future research to build upon.

### Clinical implications of the study

Our review highlights the potential for physiotherapists to incorporate mindful breathing exercises as part of a holistic management approach for individuals with major depressive disorder. Given the growing recognition of the mind–body connection in health outcomes, physiotherapists are well-positioned to deliver non-pharmacological interventions that support both psychological and physiological well-being. The studies reviewed suggest that mindful breathing can reduce anxiety, enhance autonomic regulation and improve sleep, outcomes that are closely linked to depressive symptoms and recovery. These effects are particularly relevant in inpatient and rehabilitation settings, where physiotherapists routinely engage with individuals experiencing emotional distress, reduced motivation and sleep disturbances. Teaching patients slow, diaphragmatic breathing techniques requires minimal resources, can be easily adapted to different clinical contexts, and aligns with physiotherapy’s role in promoting self-regulation and functional recovery. Moreover, breathing interventions can be integrated into exercise sessions, relaxation protocols or patient education, thereby expanding the scope of physiotherapy beyond physical rehabilitation. For patients undergoing high-stress procedures such as electroconvulsive therapy, physiotherapists can collaborate within multidisciplinary teams to prepare individuals using brief breathing-based interventions that reduce pre-procedural anxiety. Hence, incorporating mindful breathing into physiotherapy practice reinforces a biopsychosocial model of care, supports mental health recovery and offers an accessible strategy for enhancing patient engagement. As evidence continues to accumulate, training programmes and clinical guidelines should consider incorporating these techniques into the broader scope of physiotherapeutic management for mood disorders.

### Limitations of the study

Despite the valuable insights provided by the two studies included in our review, several limitations must be considered. Firstly, the small number of studies included limits the generalisability of the findings. Both studies had short follow-up durations, making it difficult to assess the long-term effectiveness of mindful breathing exercises on MDD. Secondly, the outcome measures were primarily limited to sleep quality and anxiety, neglecting broader quality of life and functional recovery aspects. Both studies also lacked blinding of participants and outcome assessors, raising concerns about expectancy effects and self-report bias. Furthermore, the participant populations were drawn from specific clinical settings, limiting the applicability of the findings to broader populations. The studies also did not explore digital or mobile delivery methods, which could increase accessibility and scalability. Lastly, the broader literature on breathing-based interventions for MDD has shown considerable variability in study quality, with many trials exhibiting a high risk of bias, which complicates the interpretation of results. Future research should address these limitations by incorporating longer follow-up periods, broader outcome measures, diverse participant populations, and exploring digital delivery methods to enhance accessibility.

## Conclusion

This systematic review provides preliminary but compelling evidence that mindful breathing exercises can effectively reduce depressive symptoms and anxiety and improve sleep quality in individuals with major depressive disorders. Although only two studies met the criteria, their findings support the use of breathing techniques as a simple and low-cost addition to standard treatment. More high-quality research is needed to confirm long-term effects, explore broader outcomes and test scalable delivery methods. Despite the limited data, our review helps clarify the current evidence and highlights key directions for future research.
